# Therapeutic Drug Monitoring of Direct Oral Anticoagulants in Patients with Extremely Low and High Body Weight—Pilot Study

**DOI:** 10.3390/jcm12154969

**Published:** 2023-07-28

**Authors:** Łukasz Wołowiec, Mateusz Kusiak, Jacek Budzyński, Anna Wołowiec, Albert Jaśniak, Michał Wiciński, Agnieszka Pedrycz-Wieczorska, Daniel Rogowicz, Grzegorz Grześk

**Affiliations:** 1 Department of Cardiology and Clinical Pharmacology, Faculty of Health Sciences, Collegium Medicum in Bydgoszcz, Nicolaus Copernicus University, 87-100 Toruń, Poland; kusmat89@gmail.com (M.K.); albertjasniak@gmail.com (A.J.); rogowicz.d@gmail.com (D.R.); g.grzesk@cm.umk.pl (G.G.); 2 Department of Vascular and Internal Diseases, Faculty of Health Sciences, Collegium Medicum in Bydgoszcz, Nicolaus Copernicus University, 87-100 Toruń, Poland; jb112233@cm.umk; 3 Department of Geriatrics, Division of Biochemistry and Biogerontology, Collegium Medicum in Bydgoszcz, Nicolaus Copernicus University, 87-100 Toruń, Poland; anna.wolowiec@cm.umk.pl; 4 Department of Pharmacology and Therapeutics, Faculty of Medicine, Collegium Medicum in Bydgoszcz, Nicolaus Copernicus University, 87-100 Toruń, Poland; michal.wicinski@cm.umk.pl; 5 Department of Histology, Embryology and Cytophysiology, Medical University of Lublin, 20-059 Lublin, Poland; agnieszka.pedrycz-wieczorska@umlub.pl

**Keywords:** direct oral anticoagulants (DOACs), atrial fibrillation, obesity, body weight, therapeutic drug monitoring

## Abstract

Phase III clinical trials for individual direct oral anticoagulants (DOACs) contained a limited representation of subjects with abnormal body weight, which were mostly limited to a BMI > 40 kg/m^2^, or body weight > 120 kg for obese subjects, and <50 kg for underweight subjects. Although low or high body weight is not a contraindication to DOACs therapy, it can significantly affect the safety and effectiveness of treatment. Due to the limited amount of clinical data on the use of DOACs in extremely abnormal weight ranges, optimal pharmacotherapy in this group of patients is a matter of controversy. The objective of this study was to evaluate the pharmacokinetics of DOAC properties in patients with abnormal body weight beyond the established cut-off points in the phase III studies for rivaroxaban, apixaban, and dabigatran. In total, 38 patients took DOACs for at least 12 months for non-valvular atrial fibrillation in 2019–2021. Blood samples were collected before the planned intake of the drug and 4 h after its administration. The determined concentrations of DOACs were statistically analyzed in relation to body weight, age, and eGFR (estimated Glomerular Filtration Rate). Among subjects taking apixaban, rivaroxaban, and dabigatran, the smallest representation of patients who achieved therapeutic concentrations were those treated with dabigatran. The population of people with abnormal body weight is a potential risk group of patients, in which some of them do not reach the therapeutic range of DOACs.

## 1. Introduction

For years, anticoagulants have been the basic group of drugs that slow down or prevent blood coagulation in various mechanisms. The general indications for anticoagulant therapy are atrial fibrillation and flutter, acute coronary syndromes, venous thromboembolism, condition after prosthetic valve replacement, stenosis of the mitral valve, pulmonary hypertension, intracardiac thrombosis, percutaneous coronary interventions, and extracorporeal circulation [1,2].

New oral anticoagulants (NOACs) are as effective as vitamin K antagonists (VKAs) in preventing stroke in patients with atrial fibrillation (AF), but they are also associated with a 50% reduction in the risk of intracerebral bleeding compared to AF patients treated with VKAs [3].

Further, NOACs are much easier for patients and healthcare professionals to use [4,5]. First, dosing of NOACs is fixed, they do not require routine International Normalized Ratio (INR) control. What is more, food interactions are less frequent with NOACs than with VKAs [6]. The intensive development of anticoagulant therapies and the discovery of new drug molecules have made other abbreviations for NOACs, such as direct oral anticoagulant (DOAC) or target-specific oral anticoagulant (TSOAC), more appropriate [2]. Based on reports of better risk/benefit profiles of DOACs compared to VKAs, DOACs have become the drug of choice and their use has gradually expanded, including in high-risk populations [7]. Pharmacotherapy with DOACs does not require routine anticoagulation monitoring because of the wide therapeutic index. Due to the establishment of the therapeutic concentration range of DOACs on the basis of a pharmacokinetic model using only healthy subjects and patients scheduled for elective orthopedic surgery taking DOACs for a short time, there are clinical situations where accurate knowledge of drug concentration may be necessary for better risk management [8]. An indication for laboratory determination of treatment effectiveness could be a suspicion of too high or low DOAC levels, i.e., assessment of the risk of side effects or confirmation of reaching the target values of the therapeutic range of the drug. This includes, but is not limited to, bleeding, treatment failure, emergency surgery, assessment before thrombolysis, DOAC overdose, renal dysfunction, and extreme body weight [9,10]. There are limited clinical data on the use of DOACs in patients with a body mass index (BMI) > 40 kg/m^2^ or body weight > 120 kg and in patients with low body weight. Phase III studies for each of the DOACs include a small subset of patients with a BMI < 18 km/m^2^. As well, obese patient populations have been mostly limited to BMI cut-offs > 35 kg/m^2^ and >100 kg body weight (dabigatran < 50 kg and >100 kg vs. rivaroxaban and apixaban < 50 kg and >120 kg) [11]. Although low or high weight is not a contraindication to the treatment of NOACs, it can significantly affect the safety and effectiveness of therapy [12,13,14].

The continued use of DOACs in groups of patients with extreme body weights raises concerns that the pharmacokinetics (PK) and/or pharmacodynamics (PD) may change. This lack of clinical data prompted the International Society on Thrombosis and Haemostasis (ISTH) to no longer recommend DOACs in patients with BMI > 40 kg/m^2^ or weight > 120 kg [11]. However, the ISTH did not make recommendations regarding the use of DOACs in underweight patients. Obesity increases the risk of atrial fibrillation (AF), possibly due to atrial conduction disorders and recurrent AF after successful ablation [15]. Moreover, patients with high body weight are at increased risk of developing venous thromboembolism (VTE) and are often treated with anticoagulants [16]. According to the ISTH recommendation, clinicians avoid initiating DOACs in patients with high body weight because they fear that increased exposure to the drug in overweight people will reduce its effectiveness. DOACs should probably be preferred in patients with low body weight, because this group is not only at higher risk of VTE than patients with medium or high body weights, but they are likely to receive a greater benefit from DOACs [17]. There are reports of a better safety profile and more effective treatment with DOACs than VKAs in patients with BMI < 18.5 kg/m^2^ (n = 36,094, 3.8-year follow-up) [18] and/or <50 kg (n = 21,589, 1.2-year follow-up) taking anticoagulants for NVAF [19]. DOACs fall into two main groups: direct Xa factor inhibitors (rivaroxaban, apixaban, edoxaban, and betrixaban), and direct thrombin inhibitors (dabigatran and argatroban) [20]. These drugs have similar clinical uses, but their pharmacokinetics differ significantly. This paper analyzes the three most commonly used DOACs.

Dabigatran reversibly binds to the active site on the thrombin molecule, preventing thrombin-dependent activation of coagulation factors. The drug is administered as a prodrug, dabigatran etexilate, which is biologically inactive and is absorbed and converted to the active form by hydrolysis. It reaches peak plasma concentrations approximately 2 h after ingestion and its absolute oral bioavailability is 6.0%. Dabigatran is not metabolized by cytochrome P450 enzymes [21]. The elimination of dabigatran is 80% renal, possibly via protein 1 (MATE1) and protein 2K (MATE2K). This may play an important role in drug clearance and drug–drug or drug–food interactions [6,22].

Rivaroxaban is 100,000 times more selective for factor Xa than other related serine proteases. The bioavailability of rivaroxaban is estimated to be >80% after oral administration and does not change when taken with food. Plasma concentrations peak at ~3 h and the half-life is 8–11 h (may be prolonged in the elderly). The anticoagulant effect of rivaroxaban wears off after 9–12 h. The drug is metabolized in the liver, partly by CYP 3A4. About 65% of the dose is excreted by the kidneys, the rest in the feces [23,24].

Apixaban is well absorbed in the gastrointestinal tract, reaches its maximum concentration 1–3 h after ingestion, binding to proteins in 90% of the dose. Apixaban has a half-life of 8–15 h (in young subjects), is metabolized in the liver and excreted 25% in the urine and 75% in the feces [23,24].

### Pharmacokinetics in Obese and Low Weight Patients

Dosing drugs in obese people causes many difficulties [25]. The recommended doses are based on pharmacokinetics determined in studies of normal weight patients, which reduces the accuracy of estimating the appropriate amount of the active substance. Due to the coexistence of dysfunctions of organs involved in the elimination of drugs (kidneys, liver) in obese patients, the decision on dosing is more complicated. In the pharmacokinetics of the drug, four processes (absorption, distribution, metabolism, and excretion) are important, which may be different in underweight, normal, and overweight patients [26].

Absorption—despite possible differences in intestinal blood flow and gastric emptying, obesity does not appear to be associated with clinically significant changes in drug absorption from the gastrointestinal tract [27]. Scientific reports are inconsistent regarding other routes of drug administration. However, most authors agree that the absorption of drugs administered subcutaneously, intradermally, or intramuscularly may be altered in obese patients due to an increased proportion of subcutaneous fat and decreased local blood flow [28].

Distribution—drug distribution depends on the amount of adipose tissue, lean mass, blood flow, and affinity for plasma proteins and tissue structures [29]. Cardiac output, and thus organ perfusion, increases with increasing body weight [30]. On the other hand, tissue blood flow is reduced in obese individuals [31]. The described changes affect the size of drug distribution in obese people. Volume of distribution (Vd) is the apparent volume into which a drug disperses in order to produce the observed plasma concentration. One of the determinants of Vd is the patient’s body fat content and the drug’s lipophilicity. If a drug readily penetrates into adipose tissue, volume of distribution may increase dramatically in obese individuals and therapeutic concentrations will be more difficult to obtain [32].

Metabolism—the liver is the most important organ responsible for drug metabolism. Hepatic metabolism is divided into phase I and phase II reactions. Obese patients have a reduced rate of metabolism by cytochrome P450 3A4 (CYP3A4) isozymes, while metabolism by uridine diphosphate glucuronosyltransferases (UGT1 and UGT2), xanthine oxidase, N-acetyltransferase, and cytochrome P450 3A4 (CYP2E1) is increased [33]. Most data indicate that phase II reactions are more pronounced in obese patients [28].

Excretion—drugs and their metabolites are mainly excreted by the kidneys. The main risk factors for chronic kidney disease are hypertension and type 2 diabetes, which are strongly associated with obesity. On the other hand, clinical trials show that renal clearance of drugs is higher in obese patients, due to increased glomerular filtration and tubular secretion, and the effect of obesity on tubular reabsorption is still not fully understood [28,33].

## 2. Aim of the Study

The main aim of the study was to evaluate the pharmacokinetic effectiveness of DOACs treatment in the population of people with high and low body weight. In order to implement the main assumption, the following specific objectives were achieved:

1. Assessment of DOACs concentration in subjects with high or low body weight in terms of concentrations desired.
2. Evaluation of the correlation between drug concentrations in the blood and body weight, age, and eGFR.
3. Evaluation of the monitored therapy used in the context of manageability risk of bleeding complications and thromboembolic events.

## 3. Materials and Methods

### 3.1. Study Population

The study group consisted of 38 Caucasian patients of the Department of Cardiology and Clinical Pharmacology at the Nicolaus Copernicus University Collegium Medicum University Hospital No. 2 in Bydgoszcz receiving DOACs for at least 12 months for NVAF in 2019–2021. The study protocol was approved by the Bioethical Committee of the Nicolaus Copernicus University in Toruń at the Collegium Medicum in Bydgoszcz (KB 243/2019) and is in line with the principles of the Declaration of Helsinki. Each patient signed an informed consent form after obtaining detailed information about the purpose and scope of the study.

The criteria for inclusion in the study were: body weight depending on the DOAC taken—dabigatran group: <50 kg or >100 kg, or BMI < 18.5 kg/m^2^ or >35 kg/m^2^, rivaroxaban group: <50 kg or >120 kg, or BMI < 18.5 kg/m^2^ or >35 kg/m^2^, and apixaban group: <50 kg or >120 kg, or BMI < 18.5 kg/m^2^ or >40 kg/m^2^, anticoagulant treatment for NVAF was in accordance with the current European Society of Cardiology (ESC) Guidelines at the time of the study. In accordance with the rules applicable in the clinic, the determination of the drug concentration is always preceded by a history taking, physical examination, laboratory tests, and, if necessary, imaging tests, and the release of the result is a discussion with the attending physician and/or the patient.

The exclusion criteria were sepsis or shock from any cause on admission to hospital, recent (<3 months) myocardial infarction or stroke, active neoplasm, autoimmune diseases, impaired liver function (INR without oral anticoagulation >1.5, or bilirubin total >1.5 mg%, or 3 times the upper limit of normal for ALT), corticosteroid therapy, decompensated diabetes mellitus requiring treatment with intravenous insulin infusion, chronic inflammatory bowel diseases, recent (<3 months) surgery, acute coronary syndrome (ACS) in the last 12 months, major bleeding in the last 12 months (included gastrointestinal bleeding requiring therapy, intracerebral hemorrhage and other life-threatening bleeding), history of venous thromboembolism, history of peripheral embolism, and/or thrombophilia. Estimated glomerular filtration rate was calculated using the Cockcroft and Gault formula [34]. A tabular justification for the above criteria is presented in Table 1.

### 3.2. Direct Oral Anticoagulant Determination

Determination of dabigatran, rivaroxaban, or apixaban concentrations consisted of drawing the patient’s venous blood 15 min before the next dose of the drug taken and 4 h after taking the drug. The doses of the drugs taken were adjusted to applicable Guidelines of the ESC [2] and in accordance with the summary of product characteristics. The standard dose of dabigatran was 2 × 150 mg, rivaroxaban 1 × 20 mg, and apixaban 2 × 5 mg. The reduced dose was 2 × 110 mg for dabigatran, 1 × 15 mg for rivaroxaban, and 2 × 2.5 mg for apixaban. Blood was collected in trisodium citrate tubes. Dabigatran concentrations were determined using the Direct Thrombin Inhibitor Assay by HemosIL cooperating with ACL TOP Family analyzers (ACL TOP 700, ACL TOP 500 CTS, and ACL TOP 300 CTS)—diagnostic tool for in vitro quantification of dabigatran in human citrate plasma. Results are given in ng/mL. Limitations in the performance of the test are hemolysis in the sample, excessive lipemia, or too high bilirubin concentration. Concentration does not affect the results hemoglobin up to 300 mg/dL, triglycerides up to 873 mg/dL, bilirubin up to 40 mg/dL, or the presence of unfractionated or low molecular weight heparin up to 2.2 IU/mL. Range concentration values given by the manufacturer are 20–2000 ng/mL. The analytical sensitivity of the method is 2 ng/mL. A dabigatran concentration of 45 ng/mL is considered low control and a concentration of 196 ng/mL is referred to as high control [1]. Rivaroxaban and apixaban concentrations were determined using the Liquid assay Anti-Xa by HemosIL. It is an automated chromogenic assay for quantification determination of unfractionated heparin (UFH), and low molecular weight heparin (LMWH). This assay is also intended to measure direct concentrations of Xa inhibitor factors, rivaroxaban and apixaban, in human citrate plasma compatible with ACL TOP Family systems in combination with HemosIL Rivaroxaban Calibrators and HemosIL Apixaban Calibrators. Results of rivaroxaban and apixaban concentrations are given in ng/mL. Rivaroxaban concentration results are not affected by hemoglobin up to 550 mg/dL, bilirubin up to 40 mg/dL, and triglycerides up to 1151 mg/dL. Apixaban concentration results are not affected by hemoglobin up to 300 mg/dL, bilirubin up to 25 mg/dL, and triglycerides up to 1156 mg/dL. The detection range of rivaroxaban concentrations stated by the manufacturer is 20–1000 ng/mL. The analytical sensitivity of the method is 10 ng/mL. A rivaroxaban concentration of 79 ng/mL is considered low control, and a concentration of 299 ng/mL is defined as high control [1]. The detection range for apixaban concentrations is 15–1000 ng/mL. The analytical sensitivity of the method is 6 ng/mL. Concentration of an apixaban amounting to 80 ng/mL is considered low control and a concentration of 322 ng/mL is referred to as high control [1]. In addition, all participants underwent the following tests: plasma creatinine concentration with calculation of the glomerular filtration rate (eGFR, calculated according to the MDRD formula), INR expressing the prothrombin time, activated partial thromboplastin time (APTT), and D-dimer and antithrombin III concentrations.

### 3.3. Direct Oral Anticoagulant Standards Adopted in This Study

In the Guidelines of the ESC [2], estimated normal plasma concentrations of dabigatran, rivaroxaban, and apixaban can be found; but these guidelines are based on results from single pharmacokinetic studies and cannot be an unquestionable model. Based on the experience of the local center and previously published papers, we can conclude that each analyzed DOAC exhibits anticoagulant effects, and thus, reduces the risk of thrombotic complications at 40 ng/mL. The optimal range is 40–200 ng/mL and the concentration above 400 ng/mL is associated with a significantly increased risk of bleeding complications [1].

### 3.4. Statistical Analysis

The comparison of the values of qualitative variables in groups was performed using the test chi-square (with Yates correction for 2 × 2 tables) or Fisher’s exact test where tables showed low expected frequencies. Comparison of variable values quantitative tests in three groups were performed using the Kruskal–Wallis test. After detection statistically significant differences, post hoc analysis was performed with Dunn’s test in order to identify statistically significant groups. Correlations between variables quantitative data were analyzed using the Spearman correlation coefficient and multiple regression method. Logistic regression was also used. In the analysis, a significance level of 0.05 was adopted. Thus, all *p*-values less than 0.05 were interpreted as evidence of significant dependencies. Sample size was calculated with the following assumptions: use of one-factorial ANOVA; 90% power (1-β) and α < 0.005. Due to the pilot dimension of the study and the small size of the research group, the calculated sample size was 103 for a comparison of single study group, 258 for regression analysis, and 58 for multiple regression. Statistical analysis was conducted using the licensed version of the statistical analysis software STATISTICA version 13.1 (TIBCO Software, Inc., Palo Alto, CA, USA, 2017).

## 4. Results

### 4.1. Clinical Characteristics

**Figure 1:**
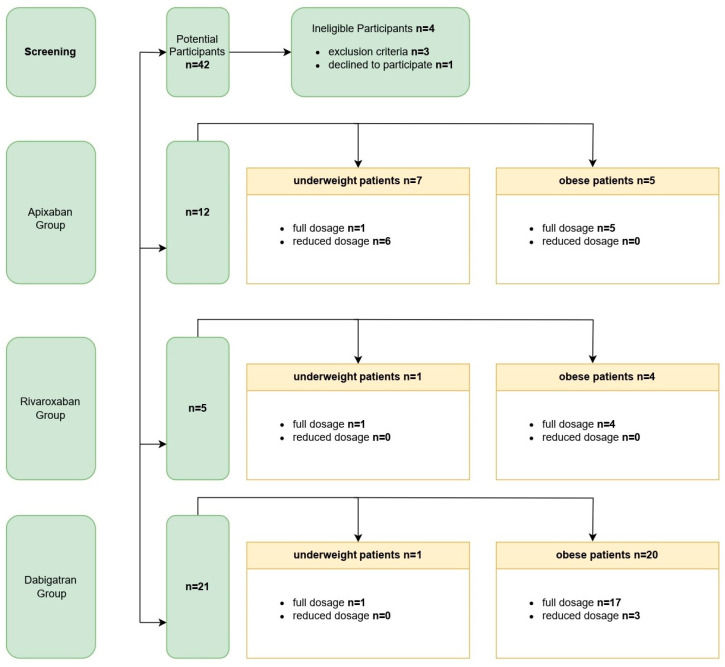
Eligibility for the study and patient affiliation to particular DOAC groups.

The study group consisted of 38 Caucasian patients receiving DOACs for NVAF. Of the 38 patients, 21 (55.26%) were treated with dabigatran, 12 (31.58%) with apixaban, and 5 (13.16%) with rivaroxaban. Among the respondents, 29 were men (76.3%), and 9 subjects were women (23.7%). In total, 29 subjects took the full dose of a given anticoagulant (76.3%), and 9 subjects took a reduced dose (23.7%). The mean age of the examined patients was 65.89 ± 13.06 years. Each of the cohorts receiving a particular drug contained a representation of patients with extreme weight ranges—apixaban group—min. body weight 40 kg, max. body weight 175 kg, dabigatran group—min. body weight 40 kg, max. body weight 146 kg, and rivaroxaban group—min. body weight 45 kg, max. body weight 160 kg. Creatinine clearance was calculated using the Cockcroft–Gault formula. Patient flow through the study and their DOAC group affiliation are presented in Figure 1. General characteristics of individual DOAC groups are presented in Table 2.

### 4.2. Assessment of DOACs Concentration in Subjects with High or Low Body Weight in Terms of Concentrations Desired

DOAC concentrations in individual groups measured 15 min before and 4 h after taking the drug are presented in Table 3. Due to the lack of differences in the concentrations of individual DOACs and due to the small size of the study group, further statistical analysis of our pilot study was preceded by dividing patients into quartiles according to weight (101, 114.126), age (58, 64.76), and GFR (51, 66.87).

### 4.3. Evaluation of the Correlation between Drug Concentrations in the Blood and Body Weight, Age, and eGFR

DOAC concentrations depending on the quartiles of the analyzed factors: weight, age, and eGFR are presented in Table 4. The collected results suggest a relationship between eGFR quartiles and DOACs concentrations both before and 4 h after taking the scheduled dose of the drug. The lowest value was obtained among participants with the highest creatinine clearance—87 mL/min. The table was prepared with the assumption of no differences between the concentrations of individual DOAC in subgroups, therefore all DOACs were analyzed together. The obtained relationships were verified below using Spearman’s rank correlation [Table 5] and multiple regression [Table 6].

In multiple regression for the drug concentration 15 min before taking the planned dose, we showed a significant and independent effect of the type of DOAC, daily dose of the drug, and eGFR. Similar analysis was performed for respective DOACs concentration 4 h after drug taking and for delta of serum DOACs concentrations, but we did not find statistically significant relationships.

### 4.4. Evaluation of the Monitored Therapy Used in the Context of Manageability Risk of Bleeding Complications and Thromboembolic Events

The analysis was performed for each DOAC separately, both before and after its administration, by comparing the number of patients in particular concentration ranges of the drug—low concentration, therapeutic concentration, and high concentration. After analyzing the percentage of patients with too low (thrombotic risk) and too high (risk of bleeding complications), we found that the comparison of the percentage of patients with low desired and too high concentration depended on the eGFR quartile only in relation to the baseline concentration. For the results presented in Table 7, there was no relationship between the type of DOAC and the achievement of the therapeutic range (*p* = 0.55). Importantly, only 60.00–76.19% of the participants reached therapeutic DOAC levels before the next dose. This result strongly supports the introduction of DOAC monitored therapy in patients with extremely abnormal body weight.

Since only 57% of participants in the dabigatran group reached the therapeutic concentration, and as many as 38% reached the high concentration of the drug, we performed a logistic regression analysis (Table 8) to assess what independently affects the achievement of the therapeutic range. For DOACs concentration 15 min before taking the planned dose of DOAC, we did not find a statistically significant equation of logistic regression (Chi2 = 0.94 *p* = 0.92), however, for DOACs concentration determined 4 h after taking the tablets, we got a statistically significant equation (Chi2 = 12.323 *p* = 0.015), in which type of DOACs treatment (apixaban, rivaroxaban, dabigatran) was an independent variable determining therapeutic level achievement, and eGFR had borderline statistical significance, as seen in Table 8.

Logistic regression showed that reaching the therapeutic concentration 4 h after drug administration depends only on the type of drug. A negative regression sign indicates that the least favorable drug is dabigatran with the highest numerical value. This is confirmed by Table 7, which show that only 57.14% of patients treated with dabigatran reached the therapeutic level after 4 h. Moreover, the multiple regression equation with variables as seen in Table 6 dedicated for dabigatran concentration 15 min before drug administration showed an R^2^ = 0.85, which means that patient’s weight, age, eGFR, DOAC’s daily dose explained 85% of the variance of dabigatran, indicating that it is the most susceptible to the influence of clinical factors on pharmacodynamics. Multiple regression equations were also obtained for the concentration at 4 h post-dose and for the delta, but they were not statistically significant.

## 5. Discussion

For patients whose concentrations fall outside the therapeutic range of current DOAC therapy guidelines, specialist center-monitored therapy may be considered [15], or alternative therapy with another DOAC or with a VKA may be possible. Such management may reduce the likelihood of thromboembolic events or life-threatening bleeding, resulting in improved safety of therapy. The distribution of drugs in the body is dependent, among other things, on the lipophilicity of drugs, which is expressed as log P, where high log P values indicate high lipophilicity [26,35]. The DOAC representatives analyzed were reported as moderately lipophilic [36] with rivaroxaban (log P 1.74 to 1.90) being the least lipophilic, followed by apixaban (log P 1.83 to 2.22) and finally dabigatran with the highest lipophilicity (log P 4.59 to 5.17) [12,35]. As a result, DOACs with higher lipophilicity should show a stronger tendency to accumulate in the adipose tissue of morbidly obese patients, and should not show this tendency in underweight patients [26]. Complementing these observations is a 2021 retrospective cohort study that included adults with NVAF or atrial flutter (AFL) receiving DOACs ≥ 12 months. Of the 233 patients analyzed, 25 experienced major and clinically significant bleeding. Patients who bled weighed 10 kg less (*p* = 0.043) than those who did not bleed. Multivariate logistic regression identified body weight (*p* = 0.048) as a significant predictor of bleeding [13]. This may have been related to increased susceptibility to DOAC side effects due to lower accumulation of the drug in adipose tissue and a tendency to achieve high drug concentrations in the blood. In addition, the individual DOACs differ in renal clearance, with the highest values observed for dabigatran [23]. The data collected by us from among the three analyzed factors: weight, age, and eGFR, indicate that the eGFR quartiles are related to the DOAC concentration both before the scheduled intake of the dose and 4 h after taking it. However, it should be noted that research shows that body weight may affect kidney function, with eGFR averaging 62% more in obese than non-obese people [33]. In our study, we observed that achieving therapeutic concentration among patients with extremely abnormal body weights is burdened with an increased risk. We noticed differences between the individual DOAC groups in achieving low, desired, and high concentrations. This raises the question of which DOAC is the most optimal for patients with abnormal body weight and whether there are different preferences in the choice of DOAC among underweight and obese people. Of all the groups participating in the study, the group receiving dabigatran achieved the lowest percentage of reaching the therapeutic concentration 4 h after taking the planned dose of the drug, and was the only one with a representation of people with high concentrations already before taking the next scheduled dose. The difficulty in obtaining therapeutic concentrations of dabigatran among patients with abnormal body weight treated with DOACs is indicated by some scientific reports. Myrthe M. A. Toorop at al. conducted a cross-sectional study in which patients taking DOAC (n = 148) with an average body weight of 88 kg (standard deviation 18 kg) gave blood samples prior to the scheduled dose of DOAC to determine drug trough concentrations, which were measured using a validated liquid chromatography–mass spectrometry/mass spectrometry assay. The univariable analysis showed a significant negative association between body weight and dabigatran concentrations (0.73 ng/mL/kg, 95% CI 135 to 0.11), and body weight was negatively associated with apixaban concentrations (0.45 ng/mL/kg, 95% CI 138 to 0.47), while results were around unity among patients taking rivaroxaban (0.02 ng/mL/kg, 95% CI 0.34 to 0.31). Researchers believe that people with high body weight may require an escalation of DOAC dosing when treated with dabigatran [12]. Another study by Lin et al. involved 46 Asian patients treated with dabigatran. Pre-dose trough concentrations of dabigatran were found to be higher in patients > 75 years of age, body weight < 60 kg, creatinine clearance (CrCl) < 50 mL/min, CHA2DS2-VASc score > 3 points, and HAS-BLED > 3 points. Multiple linear regression analysis identified body weight and serum creatinine as key predictors of trough levels (*p* = 0.003 and 0.005, respectively). They concluded that monitoring of dabigatran concentrations may be considered in patients at risk of overexposure, especially in patients with low body weight and renal insufficiency [14]. A review of the available studies indicates the potential superiority of apixaban and rivaroxaban over dabigatran in the population of patients with abnormal body weight. This is consistent with the findings of our pilot study, in which the apixaban and rivaroxaban groups achieved significantly greater therapeutic representation than dabigatran-treated subjects at 4 h post-dose. In contrast to dabigatran, no participant taking apixaban or rivaroxaban was in the high drug concentration range 15 min before the next scheduled dose, which could be associated with a potential risk of bleeding.

A 2020 study examining the pharmacokinetics of rivaroxaban in patients demonstrating extreme body weights at King’s College Hospital Anticoagulation Clinic (n = 913, min. 39 kg, max. 172 kg) showed that Cockcroft–Gault creatinine clearance was the most significant covariate affecting rivaroxaban exposure. The investigators believe that rivaroxaban can be used successfully regardless of body weight provided that renal function is satisfactory, also recommending a revision of the previously quoted ISTH [11] position regarding the non-use of DOACs in patients > 40 kg/m^2^ or weight > 120 kg and recommending rivaroxaban in the population of people that are overweight [37]. The researchers’ assumptions were confirmed in two meta-analyses from 2020 [38] and 2021 [39], which compared DOAC with warfarin in patients with morbid obesity and atrial fibrillation. Both meta-analyses showed statistical superiority in terms of safety and efficacy, not only for rivaroxaban but for all DOACs compared to warfarin. Only dabigatran showed noninferiority to warfarin in stroke or systemic embolism rate, but superiority in the safety outcome in terms of major bleeding [39].

There are more reports of a safe profile of rivaroxaban as well as apixaban [40] in populations of people with extremely abnormal body weight, while the least favorable safety and efficacy profile seems to be achieved by dabigatran [41]. A 2020 publication review analyzing the use of DOACs among patients with abnormal body weight lists rivaroxaban and apixaban as reasonable therapeutic options for patients with high body weight, and apixaban as the best choice for people with low body weight. The authors recommend avoiding dabigatran in the patient population with abnormal body weight [42]. The use of therapy monitored based on peak drug-specific anti-Xa chromogenic assay results and adjusting the dosage to body weight allowed VanderPluym et al. to achieve effective treatment with apixaban characterized by a low rate of adverse events in a diverse cardiac population of children and adolescents. In their study published in 2023 [43], over a 3-year period, 219 children with an average age of 6.8 years (0.3–19) and an average weight of 20.8 kg (4.8–160) received apixaban for a total of 50,916 patient days. Of them, 172 (79%) warranted thromboprophylaxis and 47 (21%) thrombosis treatment (with 10 arterial, 19 venous, 15 intracardiac, and 3 pulmonary). The median initial peak apixaban level was 165 ng/mL (23–474; n = 125) in the prophylaxis subgroup and 153 ng/mL (30–450; n = 33) in the treatment subgroup; dosage was adjusted in response to levels in 25% of the patients. There were four bleeding safety events (three clinically relevant non-major bleeding; one major, hemoptysis complicating empyema); the serious bleeding event rate was 2.9 per 100 patient-years of apixaban. Minor bleeding events [43] were noted in 18 patients, with an additional 2 patients having leukopenia, 1 patient having transaminitis, and 3 patients having rashes. An improvement in thrombosis was seen in 95% of the treated patients with available follow-up imaging (37/39 patients). This is an important study because the use of DOACs in pediatric cardiology is poorly defined; the dosing is based on a separate pharmacokinetic model, and despite the growing literature on the treatment and prevention of thromboembolism in children, there is still a need for evidence-based pediatric guidelines [44].

Due to the limited number of clinical trials, the lack of high-quality randomized individualized trials evaluating the optimal selection and dosing of DOACs among patients in extreme weight ranges, the conduct of optimal pharmacotherapy in these patients may be a subject of controversy. Due to the more favorable risk profile and effectiveness of DOAC treatment over current anticoagulants, [45] including VKAs, in many clinical situations [46], it seems like a good solution to consider continuation of pharmacotherapy based on DOACs with the use of monitored therapy in groups of patients at the highest risk [47,48] which include patients with abnormal weight due to not reaching therapeutic concentrations. At the same time, body weight cannot be considered in isolation but should be included as part of an integrated approach to patient care [49,50,51].

### Strengths and Limitations of the Study

A strong point of our study is the first-ever assessment of the relationship between DOAC concentrations and weight, age, gender, and especially eGFR.

The main limitation of our work is its pilot dimension and the small number of participants included in the study. The start of the study in 2019 was a time when DOACs were relatively new drugs with limited registration and reimbursement scopes. Even now, many patients, for economic reasons, decide on cheaper anticoagulants, e.g., VKA. Due to ethical reasons and the small actual population of patients meeting the inclusion and exclusion criteria at the time of screening for the study, a larger representation of the study group was difficult to achieve. Another limitation that affects the heterogeneity of the group is the use of full and reduced doses in some patients. Indications for dose reduction did not go beyond those contained in the Summary of Product Characteristics of individual DOACs. In publications concerning DOACs, there is no standardized method for calculating creatinine clearance. Different studies may utilize various methods and formulas to estimate creatinine clearance or may use different values for serum creatinine, which can affect result interpretation and DOAC dosing. The novel aspect of our study is the simultaneous statistical analysis of several DOACs performed in a European population representing both extremely low and high body weights. Our analysis included both insight into drug concentrations and its relationship with age, weight, and eGFR. Most of the available publications assess the occurrence of bleeding or thrombotic complications only in underweight or obese people, without looking at the achievement of therapeutic concentrations. As a result of the small sample size, the study did not find a significant relationship between DOAC concentrations and clinical data such as height or weight (*p* > 0.05). However, the low statistical power of the analyses (0.20–0.27) also prevented ruling out the possibility of such relationships. Nevertheless, this study could be significant for guiding future research directions aimed at personalizing DOAC dosage.

## 6. Conclusions

The population of people with abnormal body weight is a potential risk group of patients, in which some of them do not reach the therapeutic range of DOACs. Although the majority of patients achieved drug concentrations considered therapeutic, a small percentage of patients with low body weight achieved concentrations higher than desired, which may increase the risk of bleeding complications. On the contrary, some obese patients achieved DOACs concentrations lower than desired, which could increase the risk of thromboembolic events. Among subjects taking apixaban, rivaroxaban, and dabigatran, the smallest representation of patients who achieved therapeutic concentrations were those treated with dabigatran. Monitored therapy in patients with high or low body weight may be important in managing the risk of bleeding complications and in early identification of patients with insufficient treatment effectiveness. In the study population, among weight, age, and eGFR, the factor most strongly correlated with DOACs concentrations was eGFR. Due to the small study group, there is a need to conduct larger clinical trials evaluating DOACs concentrations in patients representing extreme weight ranges.

## Figures and Tables

**Table 1:** Justification of the inclusion and exclusion criteria for the study.

Inclusion Criteria	Exclusion Criteria
inclusion criteria are based on the largest registration studies for individual DOACs used in the NVAF	election of the most homogeneous group of patients with NVAF
	elimination of factors that may affect DOAC metabolism
	reduction of a number of potential drug interactions
	elimination of disease states that force dose reduction of a given DOAC or its temporary discontinuation during the last year

Abbreviations: DOAC—Direct Oral Anticoagulant; NVAF—Non-Valvular Atrial Fibrillation.

**Table 2:** Baseline characteristics of patients taking DOACs.

Parameter	Apixaban (n = 11)	Rivaroxaban (n = 5)	Dabigatran (n = 21)
weight [kg]	48.00; 45.0–124.5	48.00; 125.00–134.00	111.00; 107.00–126.00
age [years]	76.50; 59.50–81.50	76.50; 60.00–64.00	63.00; 56.00–76.00
daily dose [mg]	7.50; 5.00–10.00	7.50; 20.00–20.00	300.00; 300.00–300.00
creatinine [mg/dL]	1.13; 0.98–1.28	1.13; 0.78–1.28	1.12; 1.02–1.31
eGFR [mL/min/1.73 m^2^]	57.50; 45.00–69.50	57.50; 87.00–69.50	64.00; 51.00–81.00

Data presented as median and interquartile range (IQR); Abbreviations: Normalized Ratio; eGFR—estimated Glomerular Filtration Rate according to Cockcroft and Gault formula.

**Table 3:** Respective DOAC concentrations.

Parameter	Apixaban (n = 11)	Rivaroxaban (n = 5)	Dabigatran (n = 21)
DOAC concentration 15 min before its intake [ng/mL]	122.00; 74.00–146.00	107.00; 53.00–124.00	94.00; 60.00–132.00
DOAC concentration 4 h after its intake [ng/mL]	181.00; 161.00–245.00	234.00; 188.00–256.00	148.00; 124.00–208.00
DELTA	73.00; 46.00–95.00	134.00; 81.00–184.00	51.00; 30.00–83.00

Data presented as median and interquartile range (IQR); Abbreviations: DOAC—Direct Oral Anticoagulants. DELTA = DOAC concentration 4 h after its intake [ng/mL] − DOAC concentration 15 min before its intake [ng/mL].

**Table 4:** DOAC—interquartile range.

Parameter	DOAC Concentration	1 Quartile	2 Quartile	3 Quartile	4 Quartile	ANOVA *p*-Value
weight [kg]	15 min before drug administration	123; 101–135.6	89.5; 63–132	97.5; 65–176.5	74; 50–159	0.342
	4 h after drug administration	179; 173–250	148.5; 140–204	226.0; 111.5–251.5	188; 90–234	0.343
	DELTA	55.5; 47.5–93.0	52.5; 46–85	84.0; 26.0–156.5	79; 30–84	0.342
age [years]	15 min before drug administration	74; 55–104	85.5; 46–101.5	132; 83–160	125; 68–146	0.202
	4 h after drug administration	149; 120–243	144; 111.5–183.5	204; 141–250	195; 162–247	0.536
	DELTA	56; 46–84	53.5; 26–89.5	74; 41–112	73; 41–85	0.380
eGFR [mL/min/1.73 m^2^]	15 min before drug administration	159.5; 144.5–188.5	123.5; 92–139	77; 55–104	64; 50–99	0.002
	4 h after drug administration	243; 204–337	170.5; 142.5–226	134; 98–160	180.5; 140–256	0.018
	DELTA	68.5; 46–113.5	47.5; 30.5–90	55; 30–78	89.5; 52–184	0.258

eGFR—estimated Glomerular Filtration Rate according to Cockcroft and Gault formula; ANOVA—one-way Analysis of Variance; DOAC—Direct Oral Anticoagulants; Explanation quartiles for weight—<101, 101–113, 114–125, ≥126; age—<58, 58–63, 64–75, ≥76; eGFR—<51, 51–65, 66–86, ≥87.

**Table 5:** Spearman’s rank correlation coefficient for all DOAC (apixaban, rivaroxaban, and dabigatran) treatment groups concomitantly.

Pair of Variables	Whole Group	Apixaban	Rivaroxaban	Dabigatran
drug concentration 15 min before intake & weight	−0.173 *p* < 0.05	0.349	0.300	−0.321
drug concentration 15 min before intake & age	0.293	0.273	−0.103	0.463
drug concentration 15 min before intake & daily dose	−0.264	0.173		−0.521
drug concentration 15 min before intake & creatinine	0.440	0.491	−0.300	0.610
drug concentration 15 min before intake & eGFR	−0.622	−0.564	−0.103	−0.791
drug concentration 4 h after administration & weight	−0.165	−0.268	0.600	−0.346
drug concentration 4 h after administration & age	0.314	0.392	0.872	0.403
drug concentration 4 h after administration & daily dose	−0.330	−0.290		−0.260
drug concentration 4 h after administration & creatinine	0.220	0.671	0.500	0.365
drug concentration 4 h after administration & eGFR	−0.384	−0.797	0.872	−0.546
DELTA & weight	−0.030	−0.395	0.100	−0.175
DELTA & age	0.011	−0.291	0.821	0.109
DELTA & daily dose	−0.176	−0.231		0.230
DELTA & creatinine	−0.089	0.318	0.400	0.058
DELTA & eGFR	0.050	−0.300	0.821	−0.135

Data presented as R—Spearmen, *p* < 0.05; Abbreviations: eGFR—estimated Glomerular Filtration Rate according to Cockcroft and Gault formula.

**Table 6:** Multiple regression analysis for factors determining DOAC’s concentration 15 min before planned drug administration (F(4,32) = 4.68 *p* < 0.01; R^2^ = 0.43).

Parameter	β	β Standard Error	*p*
Intercept term			0.155
DOAC (apixaban, rivaroxaban and dabigatran)	0.84	0.40	0.043
weight (kg)	−0.17	0.16	0.282
age (years)	0.12	0.16	0.463
daily dose (mg)	−0.83	0.40	0.045
eGFR (mL/min)	−0.52	0.15	0.001

Abbreviations: eGFR—estimated Glomerular Filtration Rate according to Cockcroft and Gault formula; DOAC—Direct Oral Anticoagulant.

**Table 7:** Distribution of individual DOAC groups in three possible concentration ranges—low concentration, therapeutic concentration, high concentration.

	15 min before Taking the Planned Dose of the Drug			4 h after Taking the Drug		
	Low (%)	Therapeutic (%)	High (%)	Low (%)	Therapeutic (%)	High (%)
Apixaban	3 (27.27)	8 (72.73)	0 (0.00)	0 (0.00)	11 (91.67)	1 (8.33)
Rivaroxaban	2 (40.00)	3 (60.00)	0 (0.00)	0 (0.00)	4 (80.00)	1 (20.00)
Dabigatran	3 (14.29)	16 (79.19)	2 (9.52)	1 (4.76)	12 (57.14)	8 (38.10)
All	8	27	2	1	27	10

**Table 8:** Logistic regression model for concentration determined 4 h after taking the planned dose of DOAC.

	Chi2 = 12.323 *p* = 0.015				
	Constant B0	NOAC	Weight	Age	eGFR
rating	6.633	−1.253	−0.019	−0.051	0.044
standard error	5.007	0.587	0.019	0.044	0.023
*p*	0.194	0.04	0.334	0.247	0.061

Abbreviations: eGFR—estimated Glomerular Filtration Rate according to Cockcroft and Gault formula.

## Data Availability

All data used to support the findings of this study are available from the corresponding author upon request.
